# Balloon-Assisted Stent Advancement Technique for Stent Delivery Failure in Complex Percutaneous Coronary Intervention

**DOI:** 10.1016/j.jaccas.2026.109140

**Published:** 2026-07-06

**Authors:** Mustafa Tunahan Oz, Adnan Kaya

**Affiliations:** Department of Cardiology, Bahçeşehir University Faculty of Medicine, Istanbul, Türkiye

**Keywords:** cardiovascular disease, coronary angiography, coronary vessel anomaly, stents

## Abstract

**Objectives:**

To describe a simple balloon-based maneuver—the balloon-assisted stent advancement technique—as a guide-extension–free alternative to facilitate stent delivery.

**Key Steps:**

Advance a second guidewire distal to the target lesion. Position a small noncompliant balloon (1.5-2.0 mm) distal to the lesion and maintain complete deflation. Advance the coronary stent over the primary guidewire while using the balloon shaft as a passive mechanical rail. Withdraw the balloon after successful lesion crossing and perform standard stent deployment.

**Potential Pitfalls:**

Potential complications include distal balloon migration, interaction between the stent and balloon shaft, device crowding within the guiding catheter, and distal vessel injury. Careful balloon positioning and maintenance of complete balloon deflation are essential.

**Take-Home Messages:**

Balloon-assisted stent advancement technique may facilitate stent delivery in selected complex non-CTO coronary lesions without the need for guide-extension catheters. Proper lesion preparation and careful procedural execution are critical to ensure safe and effective use of the technique.

## Visual Summary

**Figure:**
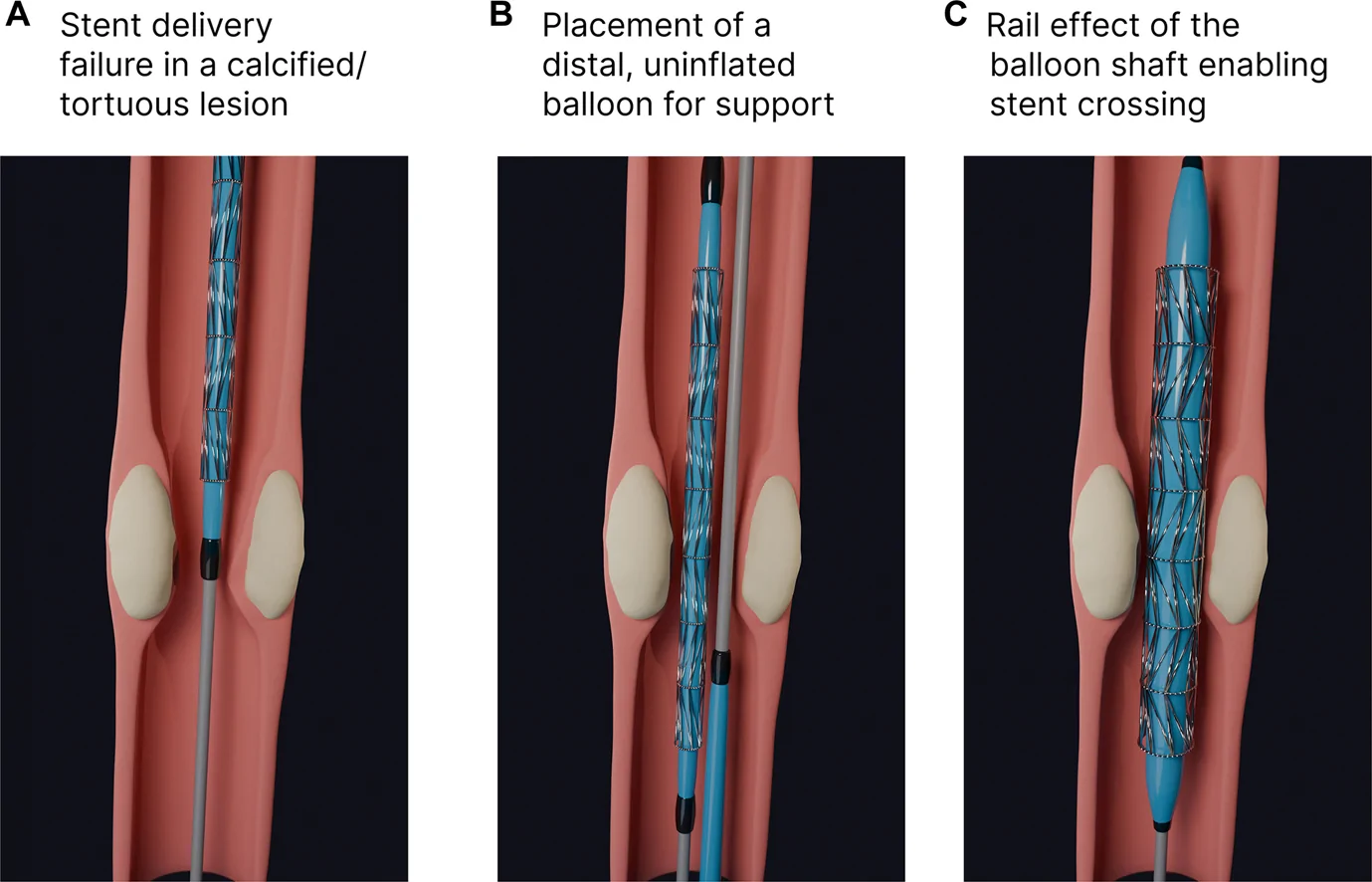

Failure of coronary stent delivery remains a relevant technical challenge during percutaneous coronary intervention (PCI), particularly in the presence of severe calcification or marked vessel tortuosity. Guide extension catheters are widely used to improve support and device deliverability; however, their use may be limited by cost, the need for additional equipment, and the potential risk of vessel trauma related to deep intubation.^1^

We describe a novel balloon-based maneuver, the balloon-assisted stent advancement technique (BASAT), which facilitates stent delivery without guide extension catheters or balloon anchoring.

## Case Summary

### Case 1

**Figure 1:**
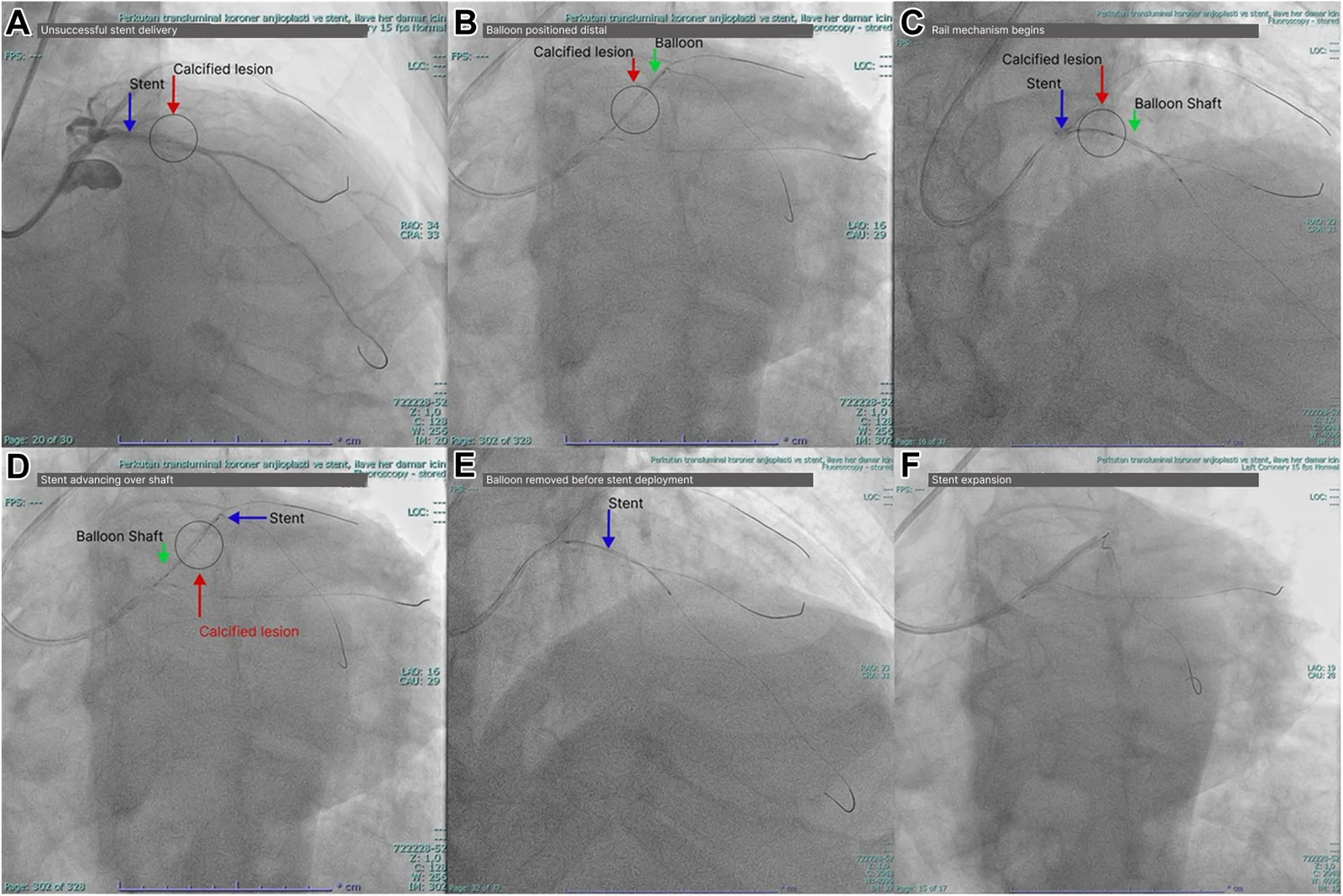
Case 1—Severely Calcified Ostial Left Anterior Descending Artery Lesion (A) Angiographic view after lesion predilatation demonstrating a heavily calcified segment with persistent resistance to stent delivery. (B–D) Stepwise demonstration of the balloon-assisted stent advancement technique rail mechanism. A completely deflated balloon positioned distal to the lesion provides a supportive rail over which the stent system is gently advanced. The stent is progressively slid over the balloon shaft, facilitating advancement across the calcified segment that previously resisted direct delivery. (E) After successful crossing of the calcified plaque, the balloon is withdrawn, and the stent is positioned precisely at the target lesion. (F) Final deployment of the stent with full expansion across the treated segment.

A patient with stable angina underwent PCI for a severely calcified ostial left anterior descending artery lesion. Despite adequate lesion preparation, stent delivery failed. BASAT enabled smooth stent advancement across the lesion, followed by successful deployment with preserved distal flow (Figure 1, Video 1).

### Case 2

**Figure 2:**
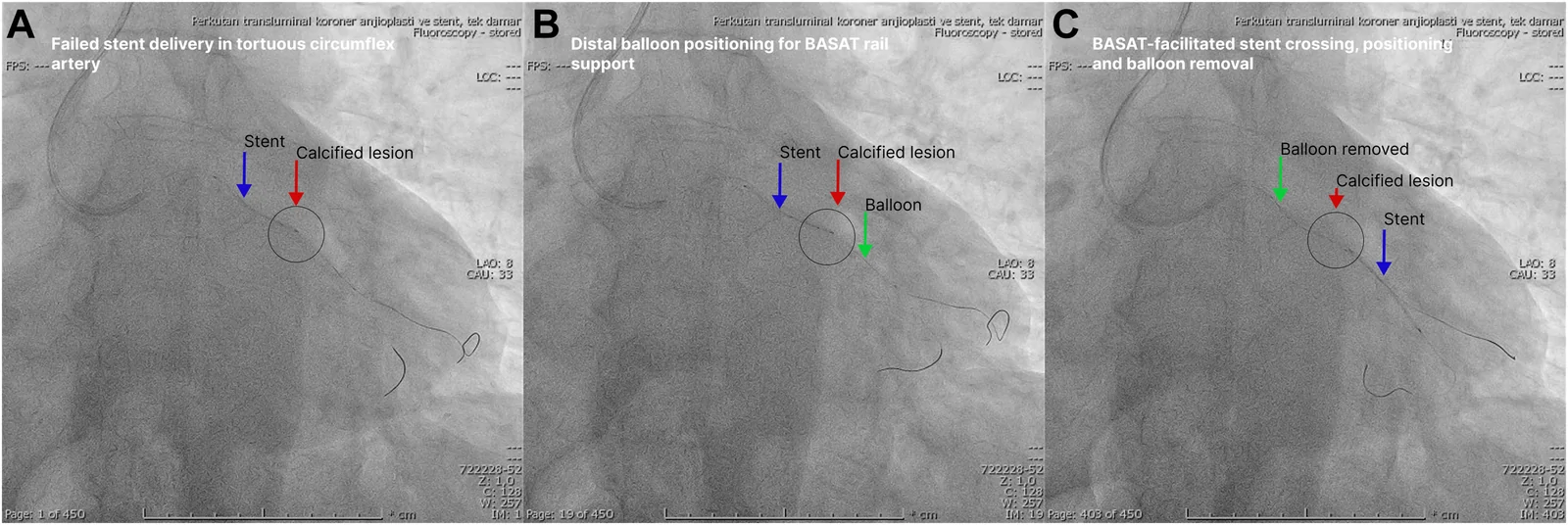
Case 2—Tortuous Circumflex Artery Anatomy (A) Coronary angiography demonstrating marked vessel tortuosity with failure of stent advancement despite standard lesion preparation and adequate guide support. (B) A noncompliant balloon is advanced distal to the target segment, providing enhanced support via the balloon shaft to facilitate device tracking across the tortuous anatomy. (C) Successful advancement of the coronary stent over the balloon shaft across the tortuous segment, allowing precise positioning prior to deployment with preserved distal coronary flow.

In a markedly tortuous circumflex artery, stent delivery was unsuccessful after standard preparation. Using BASAT, the stent crossed the lesion without the need for a guide extension catheter, achieving optimal angiographic results with TIMI flow grade 3 (Figure 2, Video 2).

## Procedural Steps

After successful wiring of the target vessel with a 0.014-inch workhorse coronary guidewire and adequate predilatation, the procedure was performed using a 6F guiding catheter providing sufficient guide support. When stent advancement remained unsuccessful despite adequate lesion preparation, the BASAT was applied.

A second coronary guidewire (buddy wire) was advanced distally. Over this wire, a small noncompliant balloon (1.5-2.0 mm) was advanced distal to the target lesion and positioned in a relatively straight vessel segment.

The balloon was kept completely uninflated (0 atm) throughout the maneuver.

**Figure 3:**
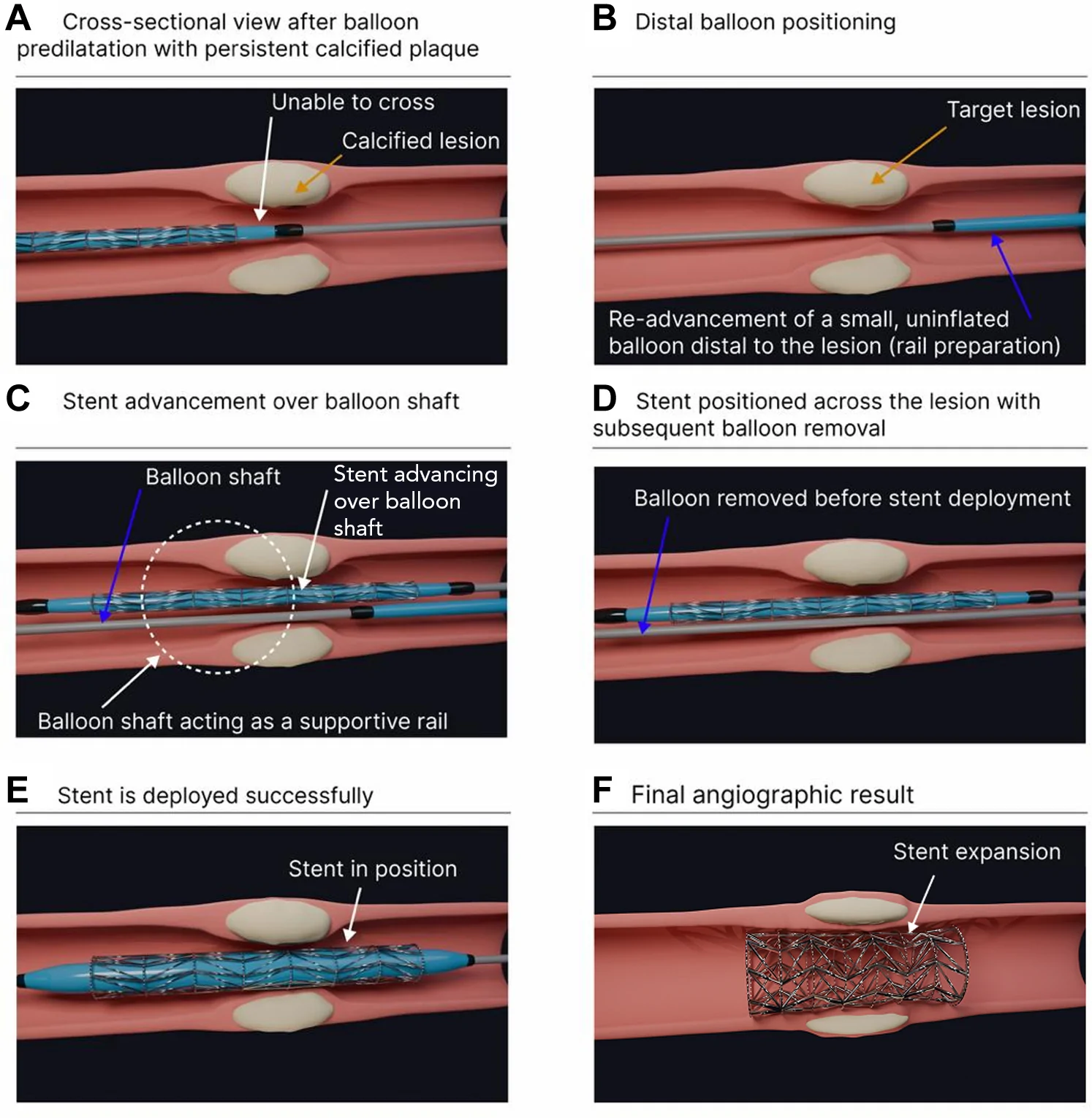
Balloon-Assisted Stent Advancement Technique (A) Failure of stent delivery across a severely calcified and tortuous coronary segment despite adequate lesion preparation, illustrating the primary procedural challenge (arrow). (B) Positioning of a noncompliant balloon distal to the target lesion within a relatively straight vessel segment to provide additional support (arrow). (C) Advancement of the stent over the shaft of the distal balloon, which functions as a supportive rail to facilitate device tracking across the resistant lesion (arrows). (D) Successful crossing and positioning of the stent across the target lesion using the BASAT rail mechanism, prior to deployment. (E) Withdrawal of the balloon catheter while maintaining stent position, preparing for deployment. (F) Final angiographic result demonstrating optimal stent expansion and restoration of luminal patency.

The coronary stent was then advanced over the primary guidewire using the balloon shaft as a passive mechanical rail, improving pushability and coaxial alignment during advancement across the resistant segment. Once the stent has crossed the lesion, the balloon is withdrawn, and standard deployment is performed (Figure 3).

### Potential Pitfalls

• Distal balloon migration
• Balloon–stent interaction
• Device crowding
• Distal vessel trauma
• Inappropriate use in CTO lesions
• Inadequate lesion preparation

## Discussion

BASAT represents a practical alternative to guide extension catheters for facilitating stent delivery in selected complex non-CTO lesions. Unlike anchor balloon or buddy balloon techniques, BASAT does not rely on balloon inflation or vessel wall fixation. Instead, it uses the balloon shaft as a passive mechanical rail, improving alignment and reducing friction at resistant segments.^2^

BASAT may be particularly useful in cases of severe vessel tortuosity or calcified lesions in which stent advancement remains difficult despite adequate lesion preparation and guide catheter support, or when advancement of a guide extension catheter is challenging.

The technique requires no additional devices, may reduce procedural cost, and avoids potential complications associated with deep guide extension intubation. BASAT should be considered after adequate lesion preparation and avoided in cases of chronic total occlusions or extremely distal vessels.

Although no complications were observed in the presented cases, several theoretical risks should be considered when applying BASAT, including distal balloon migration, interaction between the stent and balloon shaft, device crowding within the guiding catheter, and potential distal vessel injury. Careful positioning of the distal balloon and maintaining the balloon in a completely deflated state are important to minimize these risks.

Intravascular imaging with intravascular ultrasound or optical coherence tomography was not systematically performed in the presented cases; therefore, subtle vessel injury or plaque disruption cannot be completely excluded. This report is limited by the small number of illustrative cases and the absence of systematic comparison or long-term follow-up.^3^ Larger prospective studies are needed to further evaluate the reproducibility and safety of this technique.

## Conclusions

BASAT is a simple, guide-extension–free, geometry-based maneuver that may facilitate stent delivery in selected complex coronary lesions. It represents a low-cost and readily applicable adjunct to contemporary PCI practice.

**Visual Summary:**
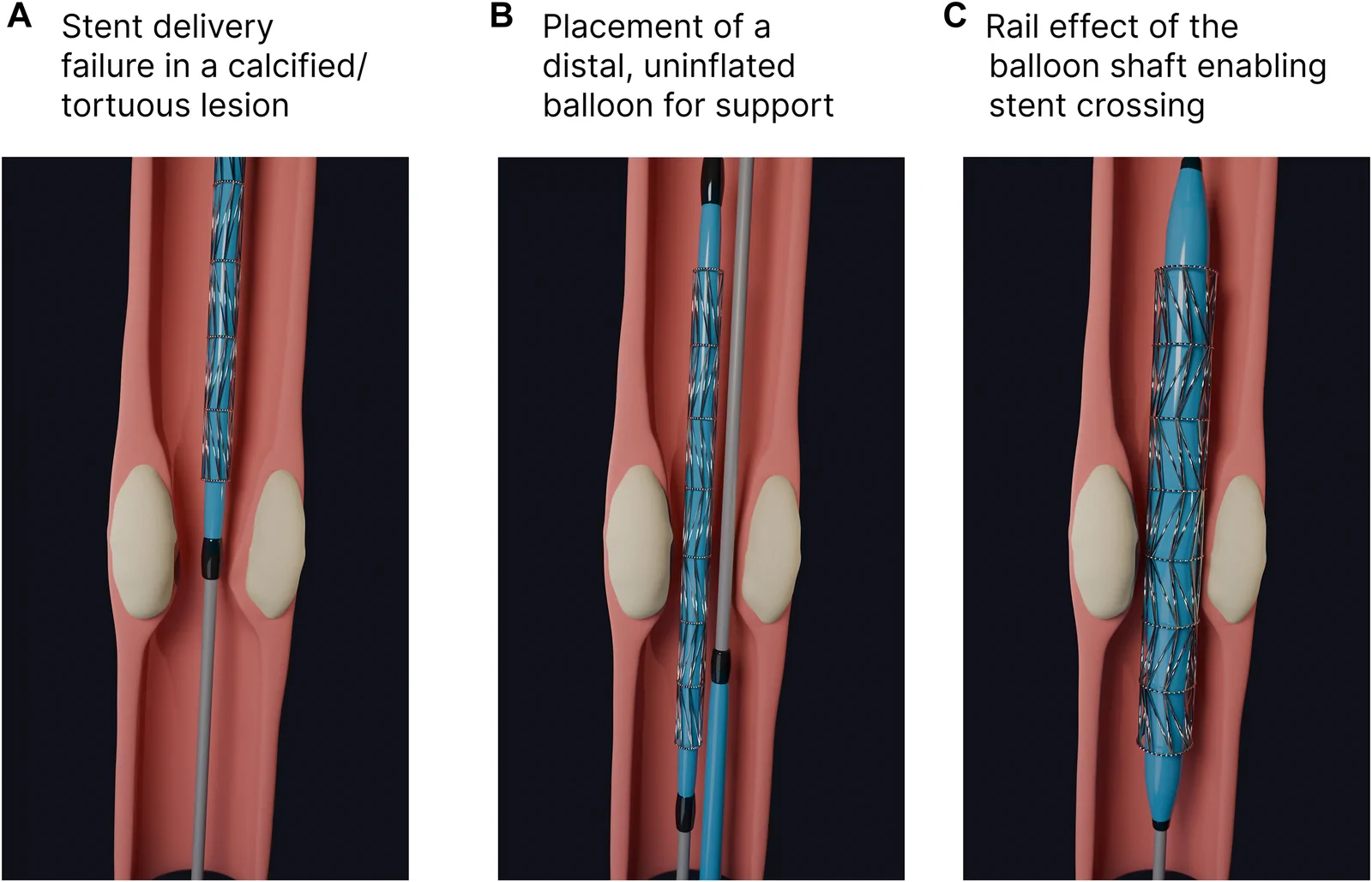
Balloon-Assisted Stent Advancement Technique (A) Failure of stent delivery due to severe calcification or vessel tortuosity despite adequate lesion preparation. (B) Advancement of a small, uninflated balloon distal to the lesion to provide additional support. (C) The balloon shaft acts as a supportive rail, improving coaxial alignment and pushability, thereby facilitating successful stent crossing.

## Funding Support and Author Disclosures

The authors have reported that they have no relationships relevant to the contents of this paper to disclose.

**Take-Home Messages**

• Balloon-assisted stent advancement technique offers a simple, low-cost, guide-extension–free solution for stent delivery failure in selected complex coronary lesions.
• When adequate lesion preparation has been achieved, balloon-assisted stent advancement technique may improve device deliverability while avoiding the additional equipment and potential complications associated with guide-extension catheters.

## Appendix

Video 1 Fluoroscopic Demonstration of the Balloon-Assisted Stent Advancement Technique, Illustrating Distal Positioning of a Noncompliant Balloon and Advancement of the Coronary Stent Over the Balloon Shaft to Facilitate Crossing of a Resistant Ostial Left Anterior Descending Artery Lesion

Video 2 Fluoroscopic Demonstration of Balloon-Assisted Stent Advancement Technique in a Markedly Tortuous Circumflex Artery, Showing Advancement of the Coronary Stent Along the Balloon Shaft to Overcome Vessel Tortuosity and Achieve Successful Stent Delivery Without the Use of a Guide Extension Catheter
